# Mechanochemistry Facilitates a Single-Crystal X-ray Structure Determination of Free Base Naloxone Anhydrate

**DOI:** 10.1021/acs.cgd.2c00831

**Published:** 2022-10-17

**Authors:** Celymar Ortiz-de León, Christopher J. Hartwick, Clara A. Stuedemann, Nicole K. Brogden, Leonard R. MacGillivray

**Affiliations:** † Department of Chemistry, University of Iowa, Iowa City, Iowa 52242, United States; ‡ Department of Pharmaceutical Sciences and Experimental Therapeutics, University of Iowa College of Pharmacy, Iowa City, Iowa 52242, United States

## Abstract

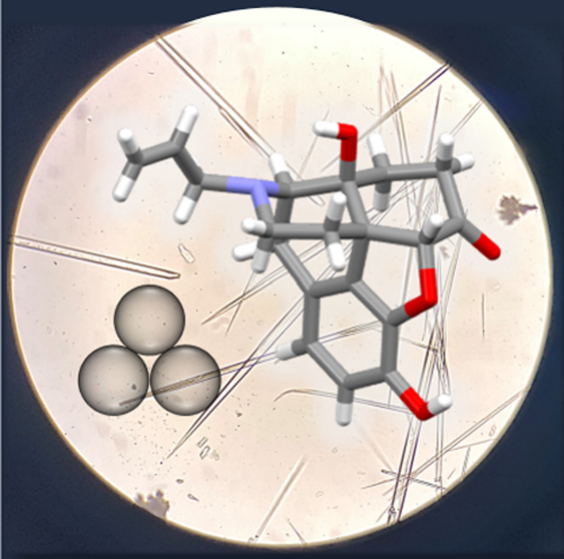

A method to obtain single crystals of the opioid antagonist naloxone in the free base form is facilitated using mechanochemistry. The application of mechanochemistry reduces the number of steps and makes single crystals readily available from solution compared to using an approach based exclusively on solution or the reported method based on sublimation. The X-ray structure confirms the structure determined using powder diffraction and provides details of hydrogen bonding.

## Abstract 2

A fresh perspective on the solid-state chemistry of naloxone is described wherein mechanochemistry in the form of ball mill grinding facilitates the first report of a single-crystal X-ray structure of free base naloxone.

## Introduction

**Scheme 1:**
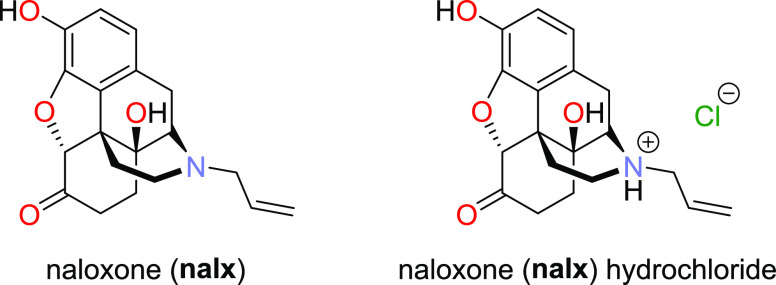
Chemical Structure of **nalx** Free Base Anhydrate and **nalx** HCl

Naloxone (**nalx**), (5α)-17-(allyl)-4,5-epoxy-3,14-dihydroxymorphinan-6-one (Scheme 1), is an active pharmaceutical ingredient (API) that serves as a highly effective antidote for opioid overdose (e.g., morphine, heroin) by antagonizing opioid receptors in the body. This API is on the World Health Organization’s (WHO) List of Essential Medicines and is currently a major societal focus owing to a declaration by the United States Department of Health and Human Services in 2017 of an ongoing opioid crisis. The API is administered intravenously, intramuscularly, subcutaneously, and intranasally in the form of a hydrochloride (HCl) salt. **nalx** is a class IV drug (low solubility and low permeability), being commercially available and administered as a salt owing to enhanced water solubility (73 mg/mL vs 1.4 mg/mL as a free base).

Despite being an API of much current scientific interest and great societal impact, a single-crystal X-ray structure determination of **nalx** in the free base form has not been published. The only related single-crystal X-ray data that have been reported pertain to the HCl dihydrate salt originally collected in 1975^1,2^ and the free base monohydrate reported in 2009.^3^ A total of three single-crystal structure determinations of the HCl salt have been described. In 2008, de Gelder et al. reported an X-ray structure of the free base of **nalx** determined from powder diffraction data.^4^ Two X-ray structures of the related API morphine (an opioid receptor agonist) determined using powder data, as both an HCl salt and a free base, were also described in the same report. An anhydrate of the HCl salt of **nalx** was also reported and determined in the same paper using powder data. Given that an appreciable amount of time and effort has been placed on studying solid phases of **nalx**, we were surprised that a single-crystal X-ray structure of the free base has not been reported.

Mechanochemistry has emerged as an important method in the study and development of pharmaceutical solid forms of APIs.^5^ Most recent efforts include applications of mechanochemistry for the syntheses of cocrystals,^6,7^ salts,^8^ and polymorphs.^9^ The generation of salt forms of APIs using mechanochemistry can be used to circumvent tedious steps of purification and isolation of solution methods, and provide a pathway to generate and discover new solid forms. While HCl salts are commonly developed and marketed forms of APIs,^10^ HCl salts have received appreciably less attention (e.g., synthesis) in the context of mechanochemistry.

Our work in pharmaceutical solid forms (i.e., hydrates, cocrystals), coupled with an awareness of societal impacts of the opioid crisis, spurred our interests to study solid forms of **nalx**. Better understanding of **nalx** solid forms and crystal structures could facilitate the development of superior dosage forms or routes of administration, which could broaden the impact of this life-saving API. In this paper, we report the first single-crystal X-ray (SCXRD) structure determination of **nalx** as a free base. In our investigation, we utilize mechanochemistry to provide ready access to single crystals of the API directly from the HCl salt. The structure that we report is generally consistent with the structure of de Gelder et al. determined using powder X-ray diffraction data yet provides additional insight into hydrogen bonding.

## Experimental Section

### Materials

Naloxone HCl anhydrate, sodium bicarbonate, and chloroform were purchased from Sigma-Aldrich. All reagents were used without further purification. Liquid-assisted grinding (LAG) experiments were carried out in an FTS-1000 shaker mill using PTFE jars (5.0 mL) and two stainless steel balls (5.0 mm diameter).

### Synthesis

#### Preparation of Naloxone Free Base (**nalx**)

**Figure 1:**
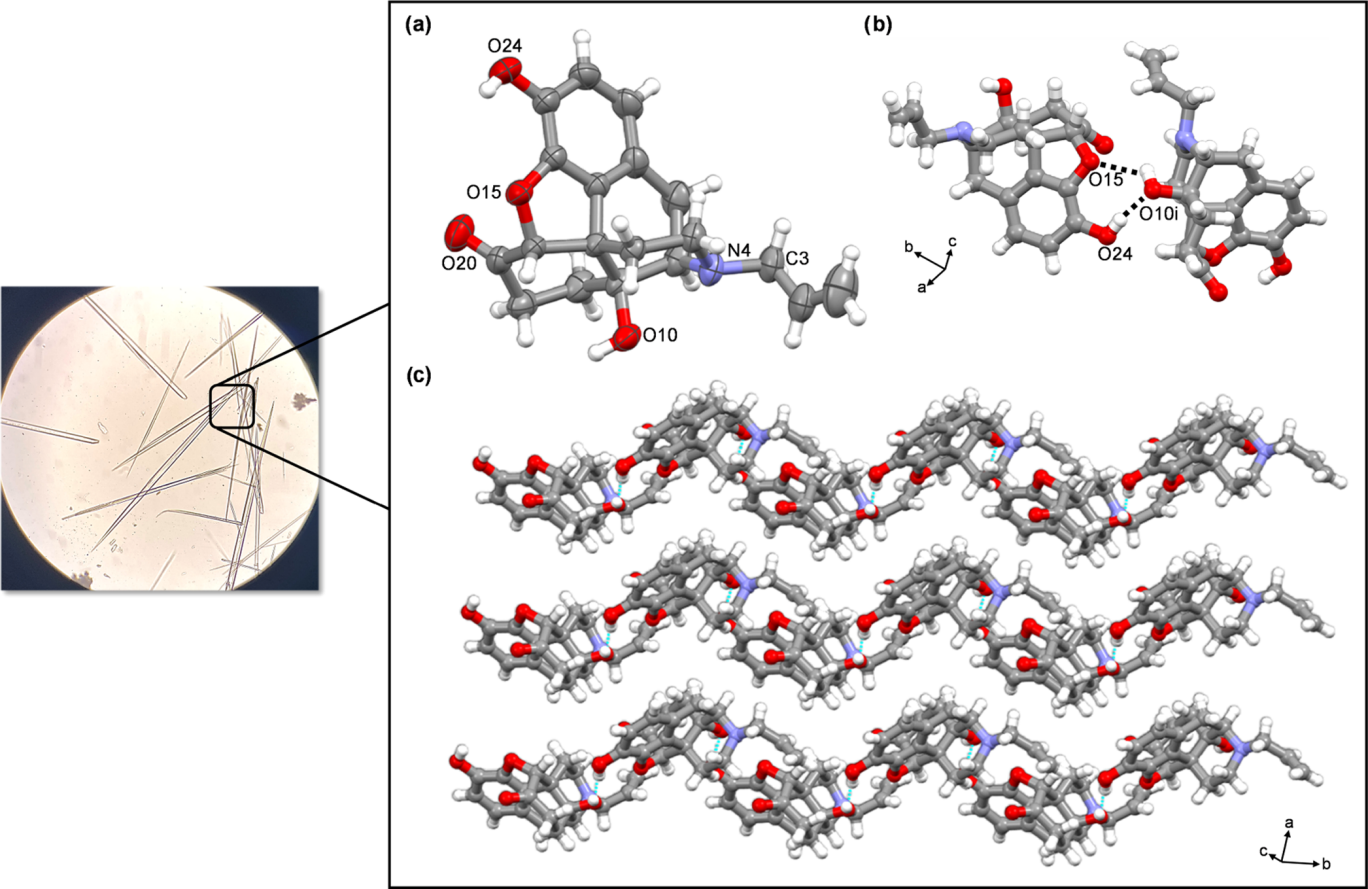
X-ray structure of **nalx**: (a) ORTEP perspective (50% probability ellipsoids); (b) O–H···H hydrogen bonds between two **nalx** molecules, symmetry codes (i) 1 – *x*, 1/2 + *y*, 1 – *z*; (ii) 1 – *x*, −1/2 + *y*, 1 – *z*; (c) layered herringbone arrangement. Inset: Needle-like crystals grown using mechanochemistry followed by evaporation from solution.

An equimolar mixture of naloxone HCl anhydrate (0.55 mmol) and NaHCO_3_ (0.55 mmol) was milled at 20 Hz for 30 min with ca. 20 μL of distilled water to form a white powder. Release of gaseous carbon dioxide from the milling apparatus was evidenced by a noticeable and rapid decrease in pressure upon removal of the screw top. The experiment can also be conducted at 20 Hz for a shorter duration (i.e., 10 min). However, higher frequency (i.e., 25 Hz) resulted in the presence of starting materials. The removal of the top was accompanied by a popping sound. The powder was suspended in chloroform (15.0 mL) and stirred for a period of 1 h at room temperature. The suspension was filtered by gravity filtration, and the solution was allowed to evaporate to near dryness to afford small colorless needle-like crystals (Figure 1). The sample was stored under ambient conditions.

### X-ray Crystallography

SCXRD data were collected on a Bruker Nonius Kappa CCD single-crystal X-ray diffractometer using Cu Kα radiation (λ = 1.54178 Å). The crystal was kept at 298 K during data collection. Data collection, initial indexing, and final cell parameter calculations were accomplished using the Bruker Apex II software suite. Multiscan absorption corrections were performed using SADABS.^11^ Structure solution and refinement were accomplished using SHELXT^12^ and SHELXL,^13^ respectively, within the Olex2^14^ graphical user interface. All nonhydrogen atoms were refined anisotropically. Hydrogen atoms associated with heteroatoms were refined via a riding model at calculated positions using HFIX commands. Figures of all structures were rendered using the CCDC Mercury software suite.^15^ The composition of the single crystal was shown to be representative of bulk material by matching the experimental PXRD pattern with the simulated from SCXRD data. PXRD data were collected at room temperature on a Bruker D8 Advance X-ray diffractometer using Cu Kα_1_ radiation (λ = 1.54056 Å) (Table 1).

**Table 1:** Summary of Crystallographic Data of **nalx**

crystal data	**nalx**^a^
empirical formula	C_19_H_21_NO_4_
formula weight (g mol^–1^)	327.37
temperature (K)	298.15
space group	*P*2_1_
*a* (Å)	7.6546(8)
*b* (Å)	12.7106(15)
*c* (Å)	8.5497(9)
α (deg)	90
β (deg)	97.290(7)
γ (deg)	90
volume (Å^3^)	825.12(16)
*Z*	2
μ (mm^–1^)	0.754
crystal size (mm^3^)	0.17 × 0.065 × 0.05
ρ_calcd_ (g cm^–3^)	1.318
*R*_1_^b^	0.0385
w**R**_2_^c^	0.1111
GooF on F^2^	1.064
flack parameter	0.00(9)
CCDC	2170599

a λ (Cu Kα) = 1.54178 Å.

b *I* ≥ 2σ(*I*).

c All data.

### Hirshfeld Surface Analysis and Fingerprint Plots

A Hirshfeld surface of **nalx** was generated using CrystalExplorer.^16^ The surface of **nalx** was mapped with the normalized contact distance *d*_norm_ over a color scale of −0.6782 au (red) to 1.5155 au (blue).

## Results and Discussion

In the report by de Gelder,^4^ single crystals of the free base **nalx** were isolated by sublimation of **nalx**·H_2_O in the presence of Ar gas. When we repeated the procedure, colorless needles accumulated on the outer tube of a cold finger apparatus. A PXRD analysis confirmed the solid as the reported structure of the anhydrate of the free base **nalx**.

The approach of de Gelder to generate the free base **nalx** involved sublimation. While sublimation is attractive for several reasons (e.g., removal of impurities), the most common method to crystallize an organic compound remains recrystallization from solution. Thus, as an alternative to sublimation, we attempted to grow single crystals of the **nalx** free base in the anhydrate form from common organic solvents (e.g., methanol, ethanol, chloroform, acetonitrile). The solution procedure that we employed involved converting the commercially available HCl anhydrate salt to the free base **nalx** in an aqueous solution of sodium bicarbonate (1:1 molar ratio), performing an extraction with chloroform and then isolating solid material using a rotary evaporator. From these experiments, the removal of the chloroform afforded a white powder. The powder, however, was determined by PXRD as the monohydrate of the free base **nalx**·H_2_O (Figure S1). When the solid was then heated for 10 min at 140 °C, the free base **nalx** anhydrate was generated according to PXRD. Attempts to grow single crystals of the free base anhydrate form, however, from saturated solutions in organic solvents invariably resulted in crystalline powders determined to be the monohydrate form of the free base **nalx**·H_2_O.

**Scheme 2:**
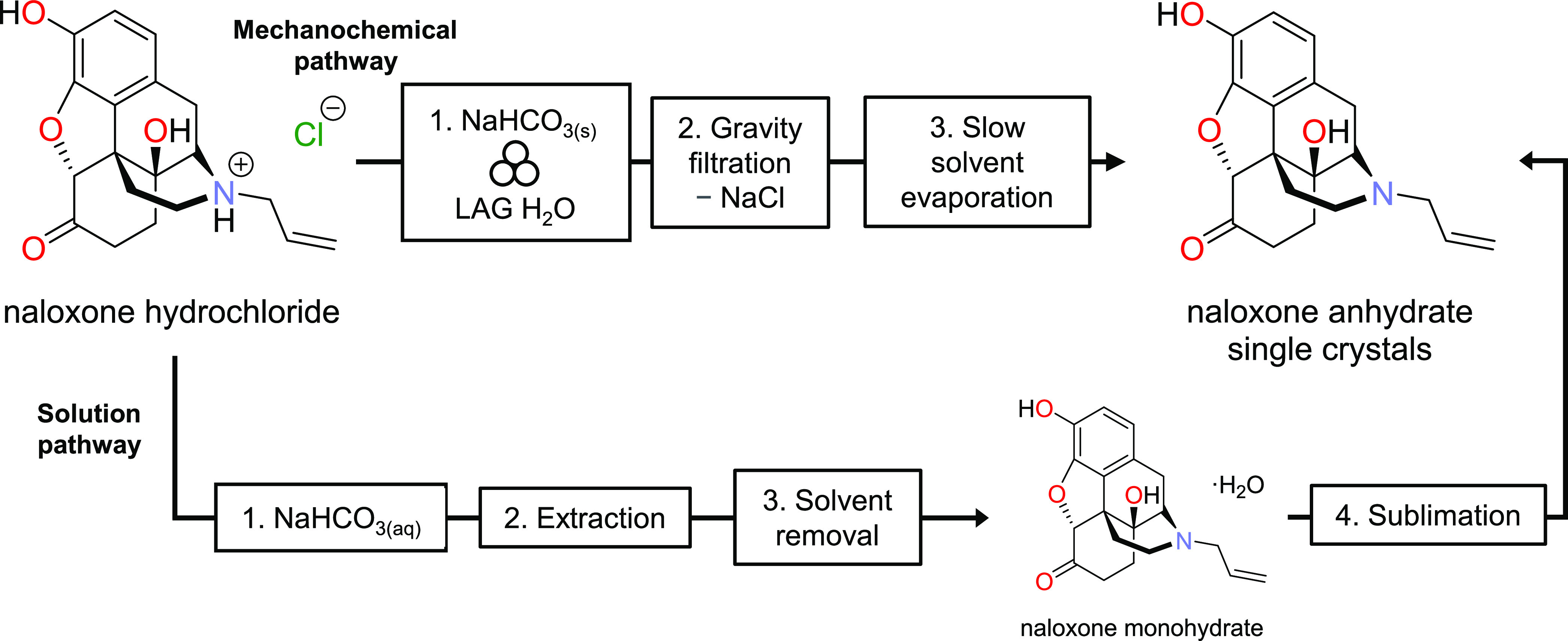
Methods to Obtain Single Crystals of the N**alx** Free Base Anhydrate Based on Mechanochemistry/Evaporation (Top) and Extraction/Sublimation (Bottom)

While attempts to generate single crystals of the free base **nalx** as an anhydrate from solution were not successful, single crystals of the anhydrate of the free base were readily generated using a combination of mechanochemistry and solution methods. Mechanochemistry has been recently used to generate crystalline phases of APIs versus solution.^17,18^ In this context, when **nalx** HCl was milled with a stoichiometric amount of sodium bicarbonate and a small amount of water (i.e., ca. 10 μL), a dry solid resulted that was determined using PXRD to be a mixture of the **nalx** free base in the anhydrate form and NaCl. The solid mixture was then stirred in chloroform at room temperature, and the NaCl was removed by gravity filtration. When the filtrate was allowed to evaporate to near dryness, single crystals of the free **nalx** anhydrate in the form of colorless needles formed after a period of two days. The process was reproducible on the gram scale using 1 g total of **nalx** HCl. For the experiment, a larger PTFE jar (15.0 mL) and additional water (10 μL) were used while maintaining the same milling frequency and time for the experiment (Scheme 2).

**Figure 2:**
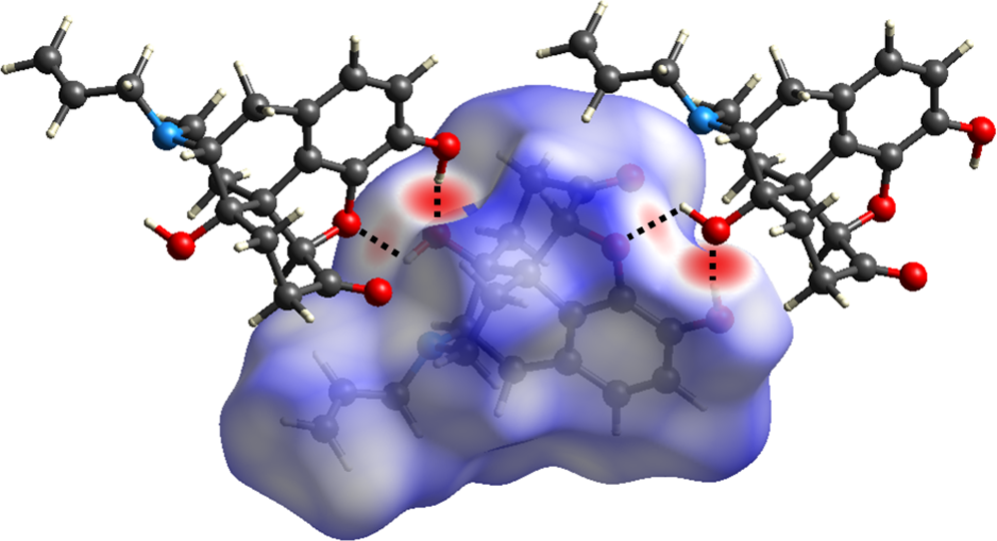
Hirshfeld surface of **nalx** mapped with the *d*_norm_ property.

A single-crystal X-ray determination of the free base **nalx** confirmed the structure as consistent with the crystal structure determined using PXRD. Specifically, **nalx** crystallizes in the monoclinic space group *P*2_1_ (Figure 1a), with the asymmetric unit consisting of one full molecule of **nalx**. The API self-assembles as one-dimensional (1D) polymers sustained by intermolecular O–H···O hydrogen bonds O(10)···O(15) 2.885(2), O(10)···O(24) 2.710(3) (Figure 1b). Adjacent 1D chains run parallel and pack in a herringbone arrangement supported by C–H···O interactions [C(3)···O(20) distance (Å): 3.576 (4)] (Figure 1c). The single-crystal structure determination enables the hydrogen bonding pattern to be identified as involving the alcoholic −OH group forming a hydrogen bond with an ethereal bridge, which was not described from the PXRD structure analysis.^19^ The intense red regions on the Hirshfeld surface of **nalx** illustrate both hydrogen bonds involving O(10)···O(15) and O(10)···O(24) (Figure 2).

In the experiments reported by de Gelder, the solution pathway to obtain the **nalx** free base generated the **nalx** monohydrate from the **nalx** HCl anhydrate. The monohydrate forms by reaction with sodium bicarbonate followed by an extraction and solvent removal. The monohydrate is then sublimed under Ar gas to yield a crystalline free base **nalx**. In contrast, the method herein yielded the **nalx** free base in three simple and straightforward steps. Milling of the **nalx** HCl anhydrate with sodium bicarbonate and a microliter amount of water directly generated the **nalx** free base with NaCl as a byproduct. Removal of NaCl using gravity filtration and evaporation of the solvent produced single crystals of the free base in the anhydrate form. The ease to generate single crystals of the **nalx** free base using the mechanochemical approach can likely be attributed to the ability of the ball milling to support quantitative and clean conversion of the HCl salt to the base (i.e., accompanied by gas release) and then subsequent clean removal of NaCl. The extraction employed in the alternative method likely provides an environment for impurities (e.g., salt forms)^20^ not to be effectively removed upon extraction that adversely impacts the formation of single crystals from solution. We note that the free base obtained by milling and then crystallization from solution can be stored under ambient conditions for extended times (e.g., 2 weeks) on the benchtop versus present in a glovebox as reported (see the Supporting Information).

## Conclusions

In this paper, we have reported a method to generate single crystals of the free base of **nalx** anhydrate using mechanochemistry. The application of mechanochemistry simplifies the process to generate and isolate the anhydrate from the commercially produced HCl salt while supporting scalability to gram amounts. The resulting single-crystal structure determination was consistent with the reported structure from PXRD. We are looking to expand the approach to other APIs and/or HCl salts.

## Supporting Information Available

Details of chemical used, PXRD data, ^1^H NMR, and fingerprint plots (PDF)
