# Supplementary material for: Mechanochemistry Facilitates a Single-Crystal X-ray Structure Determination of Free Base Naloxone Anhydrate
Source: Cryst Growth Des. 2022 Oct 17;22(11):6622–6. doi: 10.1021/acs.cgd.2c00831 (PMC9634803; doi:10.1021/acs.cgd.2c00831)

Mechanochemistry Facilitates a Single-Crystal X-ray Determination of Free Base Naloxone Anhydrate

Celymar Ortiz-de León,[a] *Christopher J. Hartwick,*[a] *Clara A. Stuedemann,*[a] *Nicole K. Brogden,*[b] *and Leonard R. MacGillivray**[a]

a Department of Chemistry, University of Iowa, Iowa City, IA 52242 United States of America

b Department of Pharmaceutical Sciences and Experimental Therapeutics, University of Iowa College of Pharmacy, Iowa City, Iowa 52242, United States of America

E-mail: len-macgillivray@uiowa.edu

Supplementary Information

1. Reagents 1
2. Powder X-ray diffraction (PXRD) measurements 2
3. Nuclear magnetic resonance (NMR) spectrum and analysis 3
4. Fingerprint plots 4

1. Reagents

Naloxone HCl anhydrate, sodium bicarbonate, and chloroform were purchased from Sigma-Aldrich. All reagents were used without further purification. Liquid-assisted grinding (LAG) experiments were carried out in an FTS-1000 shaker mill using PTFE jars.

2. Powder X-ray diffraction (PXRD) measurements

Powder X-ray diffraction (PXRD) data were collected on a Bruker D8 Advance X-ray diffractometer using CuK$_{\alpha 1}$ radiation (λ = 1.54056 Å) in the range 5–45° (scan type: coupled TwoTheta/Theta; scan mode: continuous PSD fast; step size: 0.019°) (40 kV and 30 mA).

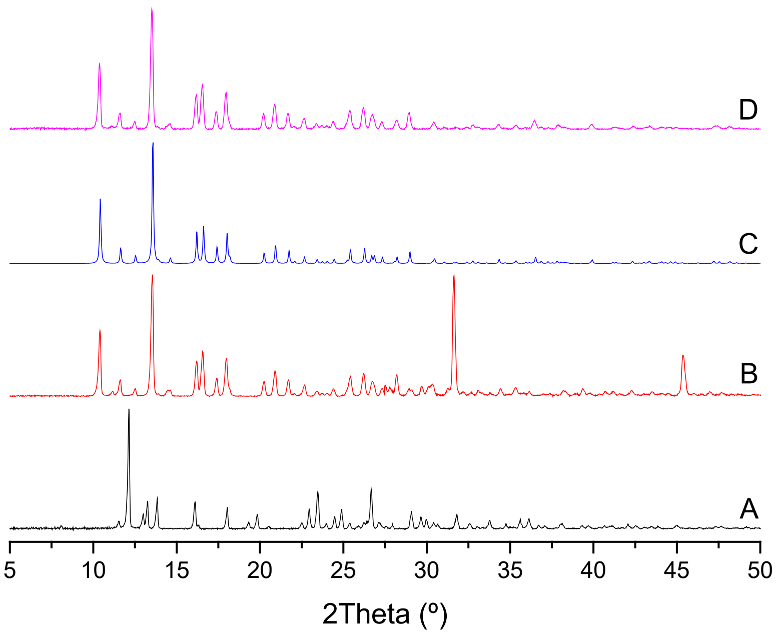

Figure S1. PXRD patterns: A) naloxone·HCl; B) **nalx** after LAG with NaCl; C) simulated from SCXRD data; and D) **nalx** after two weeks of storage.

3. Nuclear magnetic resonance (NMR) spectrum and analysis

A proton nuclear magnetic resonance (^{1}H NMR) spectrum of **nalx** was collected at room temperature on a Bruker NEO-500 NMR spectrometer operating at 500 MHz. ^{1}H NMR data are reported as follows: chemical shift (*δ*, ppm), multiplicity (s = singlet, d = doublet, dd = doublet of doublets, dt = doublet of triplets, ddd = doublet of doublet of doublets, ddt = doublet of doublet of triplets, td = triplet of doublets, m = multiplet), coupling constant(s) (*J*, Hz), and integration. Chemical shifts were referenced from the residual solvent signal of DMSO (*δ*H 2.50 ppm). The ^{1}H spectrum with resonance assignments for **nalx** is provided below (Figure S2). **^{1}H NMR** (500 MHz, DMSO-d_6): 9.03 (s, 1H), 6.42 (m, 2H), 5.72 (ddt, *J* = 16.5, 10.1, 6.3 Hz, 1H), 5.16 – 4.98 (m, 2H), 4.87 (s, 1H), 4.62 (s, 1H), 3.04 – 2.95 (m, 2H), 2.88 (d, *J* = 18.4 Hz, 1H), 2.80 –2.72 (m, 2H, overlap), 2.37 (s, 2H), 2.20 (td, *J* = 12.5, 5.1 Hz, 1H), 1.95 (dt, *J* = 14.2, 3.0 Hz, 1H), 1.87 – 1.81 (m, 1H), 1.60 (ddd, *J* = 13.3, 4.9, 3.0 Hz ,1H), 1.32 (td, *J* = 14.2, 3.0 Hz, 1H), 1.16 (dd, *J* = 12.8, 2.4 Hz, 1H).

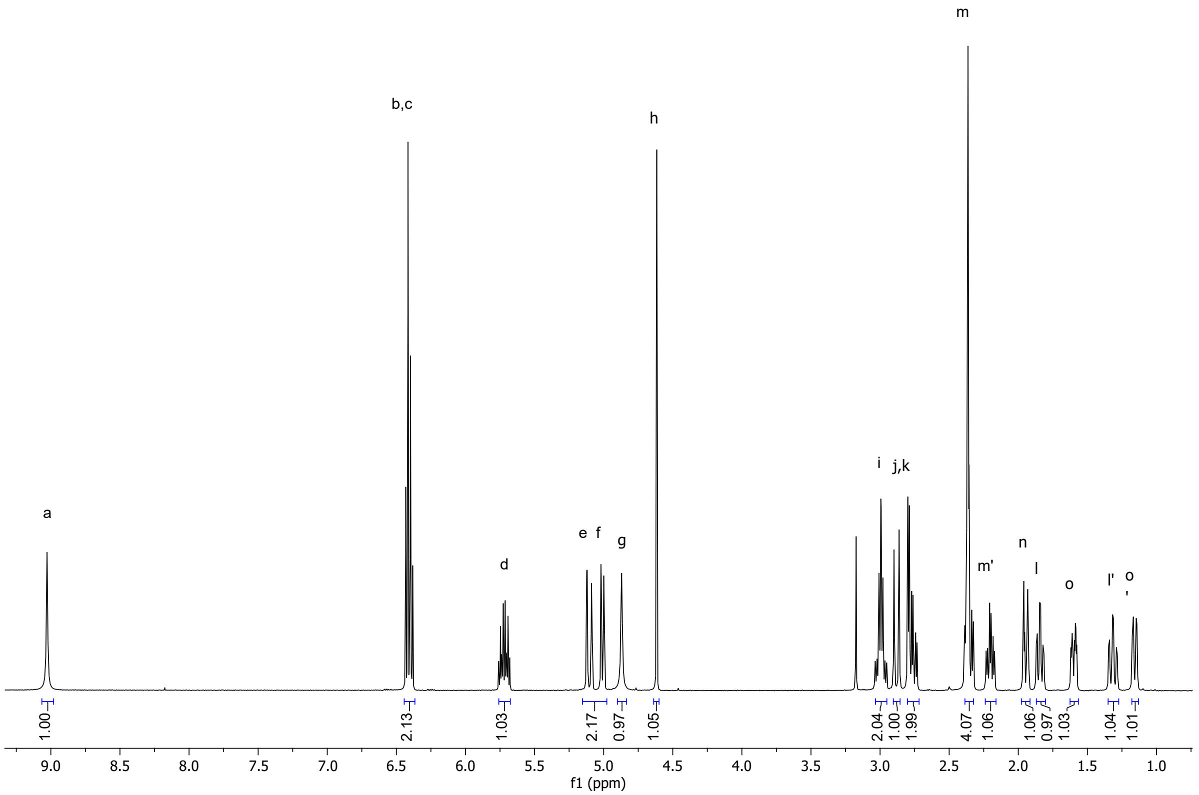

Figure S2. ^{1}H NMR (500 MHz, DMSO-d_6) spectrum of **nalx**.

4. Fingerprint plots

Table S1. Summary of the fingerprint plots of **nalx**: a) overall contacts; b) C···C contacts; c) C···O contacts; d) C···H contacts; d) O···H contacts; f) H···H contacts.

a)

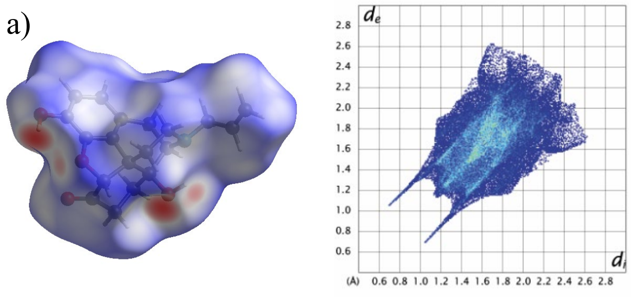

d)

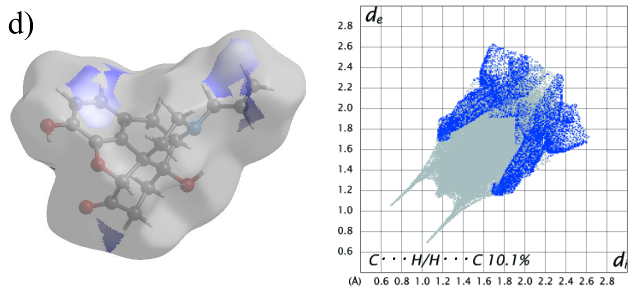

b)

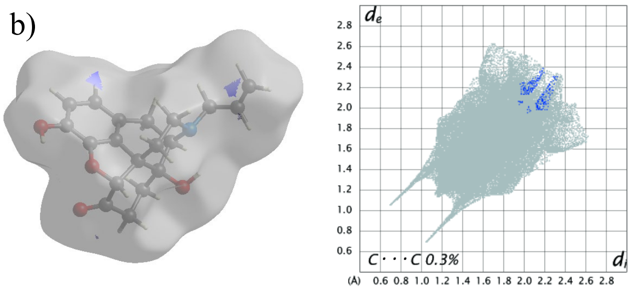

e)

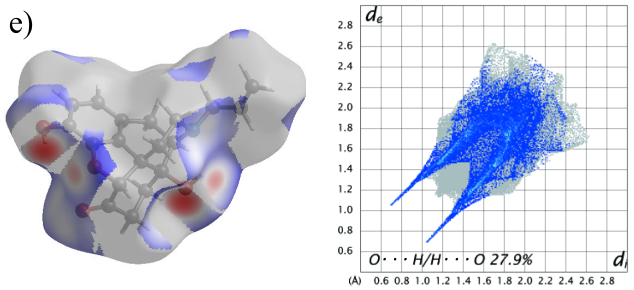

c)

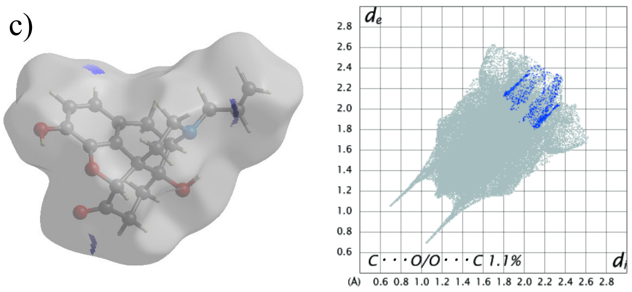

f)

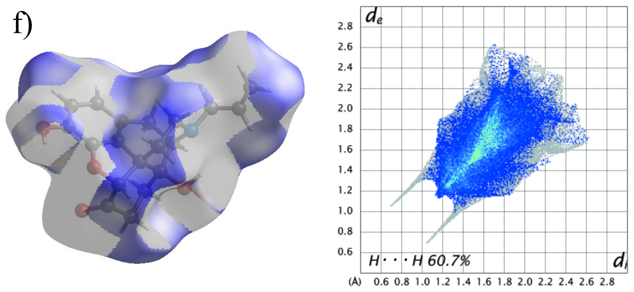

Supplement: Supplementary file 1 — cg2c00831_si_001.pdf [file cg2c00831_si_001.pdf]
